# The Ubuntu Robot: Towards a Relational Conceptual Framework for Intercultural Robotics

**DOI:** 10.1007/s11948-022-00370-9

**Published:** 2022-03-29

**Authors:** Mark Coeckelbergh

**Affiliations:** Department of Philosophy, University of Vienna, Universitätsstrasse 7(NIG), 1010 Vienna, Austria

**Keywords:** Culturally sustainable robotics, Cultural robotics, Social robotics, Wittgenstein, Ubuntu, Cultural change

## Abstract

Recently there has been more attention to the cultural aspects of social robots. This paper contributes to this effort by offering a philosophical, in particular Wittgensteinian framework for conceptualizing in what sense and how robots are related to culture and by exploring what it would mean to create an “Ubuntu Robot”. In addition, the paper gestures towards a more culturally diverse and more relational approach to social robotics and emphasizes the role technology can play in addressing the challenges of modernity and in assisting cultural change: it argues that robots can help us to engage in cultural dialogue, reflect on our own culture, and change how we do things. In this way, the paper contributes to the growing literature on cross-cultural approaches to social robotics.

## Introduction

In the past decade there has been more attention to the cultural aspects of social robots, to the extent that there is already the concept ‘cultural robotics’. For example, Samani et al., (2013) have investigated that concept; they show how cultural values impact the development of robots and even how robots themselves may hold values and create culture. They argue that robots can only contribute to the sustainability of cultural practices if they are ‘designed and used as informed by specific cultural contexts, adapted and used by human agents both as a product and a medium and eventually manifest as cultural consequences for the society in which human agents function.’ (Samani et al., 2013: 6) Following up on this work, Dunstan et al., (2016) talk in their volume on ‘cultural robotics’ about social robots as participants and creators of culture. Robots do not only work as servant or worker on an assembly line, but also produce material and non-material culture, for example when they support human creativity or are used to explore visions of what the future could be. (9) Furthermore, in science and technology studies, Sabanovic has done work on robots in different social and cultural contexts. For example, she has analyzed the co-construction of robotics and culture in Japan through the discourse and practices of robotics researchers, detecting a ‘relational’ approach to robot design in Japan (Sabanovic, 2014) that is based on a culturally specific, relational view of the self (355–356). This interest in relational views of the self is in line with Robertson, (2010), who in her paper on the gendering of humanoid robots mentions the work of the Japanese philosopher Kitaro Nishida and its influence on robotics. And the recent Springer book series ‘Social and Cultural Studies of Robots and AI’, edited by Kathleen Richardson and Teresa Heffernan, testifies to the current interest in cultural approaches to robotics.

However, in *philosophy* of technology and computer ethics, (inter)cultural perspectives are anything but mainstream, and in philosophical approaches to robot ethics little attention has been paid to conceptualizing the precise ways in which robots are related to culture. Often a Western perspective has been assumed but not made explicit, let alone critically discussed or overcome. This is now changing. For example, while in computer ethics and information ethics Charles Ess and Rafael Capurro already did seminal work on cultural approaches (consider for instance Capurro, 2008 and Ess, 2007), today this topic is receiving more attention than ever. For example, there is now a special issue in *JICES* devoted to ‘Interdisciplinary dialogues on the social and ethical dimensions of digital technologies’ (Ess, 2021: 313) that recognizes the challenge of doing computer ethics in the light of the diversity of global cultural norms (315). In robot ethics, the *Robophilosophy* conference series has always paid attention to the cultural dimension of robotics. An interesting recent effort was work in the context of the recent 2020 conference, which had the explicit theme ‘Culturally Sustainable Social Robotics” (Nørskov, Seibt and Quick, 2020), and which included contributions on the construction of culture in social robotics, on robots as icons (Sparrow, 2020), robots as empowerment technology, and the performance of identity. There also has been attention to Confucian approaches to robot ethics. For example, based on a relational and social view of the person and acknowledging the importance of rituals and norms rooted in historical traditions and cultural contexts, Qin Zhu and colleagues have proposed a robotics that enables the growth of virtues on the part of human teammates; the robot is supposed to contribute to the moral development of the human teammate (Zhu, Williams and Wen 2019). Recently, they have argued for a Confucian role-based and relational ethical framework to designing morally capable robots, and more generally for a pluralist ethics that is sensitive to diverse value systems in a global context (Zhu et al., 2021). This interest in Confucianism may be inspired by efforts of philosophy of technology pioneers such as Carl Mitcham to seek intercultural, in particular Western-Chinese exchanges, next to more general philosophical discussions about Confucian ethics, for example those by Roger Ames and Henry Rosemont (e.g., Ames & Rosemont, 2011). Yet despite these seminal contributions, more work is to be welcomed on conceptualizing the relation between robots and culture from a philosophical point of view, and on increasing the diversity of intercultural perspectives and exchanges. Doing cultural robotics is not just about a dialogue between East and West; there are many other regions and cultures we can learn from. This includes perspectives from Africa and African philosophy. For example, Capurro, (2007) already talked about Ubuntu in a keynote on information ethics ‘from and for Africa’, and Mhlambi, (2020) has proposed to use Ubuntu in order to arrive at a more relational framework for artificial intelligence governance. However, this particular direction in intercultural ethics of technology has not received wide attention, and certainly not in social robotics. African philosophical perspectives are usually ignored.

This paper aims to fill these gaps and contribute to this project of cultural robotics, including culturally sustainable social robotics, by offering a new, *philosophical* framework to think about how robots are, or can be, cultural at all, and by exploring what it would mean to talk about the “Ubuntu” robot. The latter helps to further thematize the contrast between more relational and collective societies and sensitivities, such as those represented under the umbrella concept of “Ubuntu”, and the comparatively more individualistic societies and sensibilities of “the West”. It also contributes to reflection on the meaning of technology and communication.

First, I will propose a conceptualization of the relation between social robots and culture by using a roughly Wittgensteinian approach that, in analogy to Wittgenstein’s view of language and meaning, puts the meaning and use of robots in the context of activities, games, and a form of life. This draws on my work on ‘Technology Games’ (Coeckelbergh, 2018) but now investigates the implications for an intercultural perspective.

Second, this paper explores an exchange with African philosophy, in particular Ubuntu, in order to increase the diversity of intercultural perspectives–on robotics and on philosophy of technology in general–and with the explicit aim of contributing to a more relational perspective on robotics and human being and to emphasize the role technology can play in coping with the challenges of modernity–in Africa and elsewhere.

Note that using a non-Western perspective as a Western person is always somewhat suspect; there is the danger of (illegitimate, unjust, neocolonial) appropriation, including the danger of simplification and (mis)interpretation only through Western eyes, offering a one-dimensional and romanticized view of the other culture. In this case there is the risk of kind of “Africanism,” in analogy with what Edward Said, (1978) called ‘orientalism’. For example, there is the risk of seeing African philosophy or Ubuntu as a homogeneous culture or as an ontological fixed thing (Graness, 2016), an essence. This has even happened in African philosophy itself. The philosopher Kwasi Wiredu, originally from what is now Ghana, has argued that conceptual decolonization is needed (Wiredu, 2002): a decolonization not only of countries but also of thinking. A decolonization of the mind.

These are real dangers. However, if we are really interested in intercultural work, these risks should not keep us from exploring and interpreting meanings from other cultures. Otherwise, intercultural dialogue, and in the end culturally sustainable robotics, are rendered impossible, and that would be equally problematic and irresponsible. Intercultural approaches are risky. But colonial and imperialistic thinking is avoidable and should be avoided. One way to do that is recognizing plurality and hybridity, both descriptively and normatively. Wiredu writes: ‘It seems to me likely that any African synthesis for modern living will include indigenous and Western elements, as well, perhaps, as some from the East.’ (54–55) Note also that Ubuntu is itself an umbrella term for a diversity of practices, values, and cultures in different geographical locations in Africa and beyond. For example, Mugumbate and Chereni, (2020, vi) define Ubuntu as ‘a collection of values and practices,’ the nuances of which ‘vary across different ethnic groups’, but which have in common that they see human beings as part of a larger ‘relational, communal, societal, environmental and spiritual world’. The term is also expressed differently in different African communities and languages, for example muthu in Botswana and bantu in Congo and Rwanda. However, recognizing this diversity should not keep us from working with a number of general claims about Ubuntu for the purpose of this particular project, especially when these claims have been made by African scholars rather than, say, Western anthropologists. That being said, it remains a unique challenge to translate these claims into the Western cultural context, and those who live and think within a Western context have to take up this challenge. And more needs to be said about plurality and pluralism.

Philosophically, there are at least two ways of answering the problem of intercultural translation. One is to search for universality, to look for a common language, a common logos; another is to go in a pluralist direction, as for example Raimon Panikkar did in his hermeneutics, which stresses incompatibility of different cultural views and at the same time wants a pluralism that enables love and harmony (Panikar, 1979; see also Min, 2010). The challenge is to avoid homogenization and preserve differences, but at the same time show how intercultural dialogue is possible. Panikkar rejected universalism as hegemonic and instead defended ontological pluralism: being itself is plural. He argued that intercultural dialogue is about questioning ourselves and our culture, in particular about discovering not only the myths of our dialogue partners but also our own myths, our horizon which we are not aware of. Moreover, based on Panikkar’s work on intrareligious dialogue, one could add that in such a dialogue we also discover the relational nature of reality itself. The dialogue is thus not only “inter” but also “intra” in at least two senses: (1) we discover ourselves and our culture, and (2) we discover that, in a deeper sense, we are already in dialogue (Panikkar, 1999, xix). The same could be said for intercultural dialogue, which is also always an intracultural dialogue: it is an invitation to reflect on ourselves and reveal our own myths, and ultimately it is an invitation to reflect on the nature of reality. For Panikkar, that means realizing the fundamental relationality of self and world: ‘Man is not an individual but a person, that is, a set of relationships’ (24). Before the actual dialogue, we are already interrelated.^1^ This “intra” aspect of intercultural work, in thinking about social robotics and elsewhere, is perhaps the *really* “risky” aspect and ultimately challenge: we might get to know something fundamental about ourselves, our culture, and the world. Are we prepared to go that way? And how easy or difficult is it to change a culture?

This paper explores a deep relational perspective by drawing on Ubuntu, contributes to theorizing the way we and our *technologies* are always already on a cultural horizon, and suggests that if cultural dialogue is especially about learning something about ourselves and our culture, as Panikkar argued, and about social and cultural change (an aspect I am also particularly interested in), then robots can assist with that.

Let me start by conceptualizing the relation between social robots and culture by drawing on the philosophical work of Ludwig Wittgenstein.

## In What Sense is Social Robotics Cultural? A (Roughly) Wittgensteinian Answer

According to Wittgenstein’s view of language in the *Philosophical Investigations* (Wittgenstein, 1953), the meaning of words is not linked to word-objects but depends on how we use them and in which context. In particular, Wittgenstein argues that our use of language is related to our activities and games, for example giving orders, acting, etc. He calls that which language is woven into a ‘language-game’ (1953: §7, 9e), which has its own rules and is in turn part of a larger whole, the way we do things. Wittgenstein calls this a ‘form of life’ (§19, 11e). This could be interpreted as saying that our use of language is part of a “culture”, but then not culture as some reified, externalized thing but something that is living in our use of language and in our activities and games. Culture is about ways of doing, in particular about *how we do things (here)*. Culture is not a thing but is performed. It is also social and public. Wittgenstein famously argued that there is no such thing as a private language (§243, 95e). Culture is not just about you and me; it is about *us*. Culture is relational.

Technological artefacts, I have argued (Coeckelbergh, 2018), should be understood the way Wittgenstein understands words: their meaning is always related to their use, to the activities and games and to the form of life in which it is embedded and to which it contributes. This gives us two aspects (or rather directions) of the relation between technology and culture, which also appear already to some extent in the above mentioned reflections on robotics and culture. On the one hand, technology is embedded in an existing cultural grammar, in a particular game and a particular form of life (Coeckelbergh, 2018). On the other hand, technology also contributes to that culture and form of life; it can change the game. It does this not on its own but as it is performed in a social context.^2^

What does this Wittgensteinian approach mean for social robotics? Social robots are like Wittgenstein’s words or like the technologies I talked about. On the one hand, to be culturally sustainable, they must be embedded in an existing game and form of life. For example, a robot that serves coffee will have to follow the rules of this game in a particular cultural context. If it fails at playing the relevant game, it will be seen by people of that particular culture not only as culturally unsustainable but as simply failing the task, as not functioning as a social robot. Thus, the problem is not only one of identity, belonging, and representation–people often want to have a sense of cultural ownership of the artefacts they use and may feel offended when the social robot as artefact does not represent or misrepresents their culture–but also of functioning in a particular cultural context; the criterion is a pragmatic one: success. Social robots need to be able to play the relevant game(s) well. On the other hand, in principle social robots could also be designed to change the game, for example they could do things differently than expected and thereby introduce a new, creative way of what it means to have a coffee. In that case, they will not be seen as failing but as contributing to cultural change. For instance, the robot may introduce a new coffee ritual, thereby adapting and changing the game of coffee drinking. Eventually, this may contribute to slight changes in the form of life.

Cultural change is usually not radical. While during the past decades digital technologies have dramatically, relatively rapidly, and perhaps irreversibly transformed society and culture, this is an exception, and especially when it comes to *intended* cultural change by means of the use of *words* (rather than technologies), there is usually a lot of resistance to changing *how we do things*. Forms of life tend to change slowly, and in order to meet acceptance, trust, interest, and appreciation, there will still need to be at least a reference to the older form of life and the old games (which thus also facilitates ownership and identification with the new way of doing–the new game and technology are then seen as constituting a new, creative way of promoting the same cultural identity). And even then, there is no guarantee that cultural change will happen. The outcome of processes of identification but also *communication* is uncertain. A particular rhetoric might have no effect. Technologies are also not necessarily accepted. For example, robots introduced in religious practices might well be culturally embedded, but can still meet resistance within the relevant communities (see forthcoming work by Balle and Ess). Processes of adaptation and negotiation then may or may not lead to a change of the game and, eventually, change of the form of life.

Culturally sustainable robotics for social robotics, then, means the development and use of robots that are either properly embedded in the specific game and particular form of life they are meant to function in, and/or that help us to change those games and that form of life in an interesting or better way. This twofold task corresponds to the descriptions of cultural robotics mentioned earlier: the robots have to be integrated in our culture, but they can also creatively contribute to (changing) that culture.

Now one way such game changes and cultural changes are possible is through dialogue with other traditions, which, as we saw, helps in the first place to better understand one’s own culture and tradition (and thus oneself). Such a better understanding can then help to change things. The next section explores what it would mean to use or develop an “Ubuntu robot” compared to, and different from, a Western one. Moreover, the specifics of Ubuntu philosophy will also enable me to elaborate the social and relational aspect of technology (and human beings!) already introduced by my interpretations and applications of Pannikar and Wittgenstein, and to stress that technology can play a role in cultural change and in understanding ourselves.

## The Ubuntu Robot: Towards a More Diverse and More Relational Perspective

Ubuntu is an African philosophy that is relational *par excellence*: it teaches that one can only be human through other people. I am because you are. As the Kenyan theologian Mbiti (1970) put it: ‘I am, because we are; and since we are, therefore I am’. (141) This formula fundamentally moves away from the Cartesian *cogito ergo sum*. Persons are not individuals in the Western sense but exist foremost as participants in their family, group, and community. Persons are part of a network of relationships (Ogude, 2018). This does not mean that individuality does not exist, or that there are no selfish people in African communities (Ogude et al., 2019). But in Ubuntu philosophy and culture, individuality itself is understood in a relational way. Mbiti writes: ‘Only in terms of other people does the individual become conscious of his own being, his own duties, his privileges and responsibilities towards himself and towards other people.’ (141) Human beings are deeply social and relational. We own our existence to others; we are part of the whole. The individual does not come first but is created, produced by the community and is dependent on that community from birth to death. And that community itself contains both the living and the dead. After individual death, the “we” continues to exist. The “we” provides some security in an insecure world (189). As a political philosophy, Ubuntu is about solidarity and distribution of wealth within the community. It is a form of communitarianism. Community members create strong social and cultural bonds towards one another that allow a sense of belonging, of being valued; and responsibility towards one another. This suggests a sense of obligation towards not just oneself but towards one’s community (Tschaepe, 2014).

Like modern societies, traditional communities in Africa have their own rites and regulations. With Wittgenstein we might say: their own games and their own form of life. “Individuals” are born into that form of life. These rules constrain, but they also enable and support. Mbiti describes what happens when people move to the city and have to adapt to, or find, a new form of life in a context of individualism, without the traditional solidarity. (292–293) There is confusion and alienation. Yet tensions between Western individualism and African traditional cultures, including Ubuntu world views and similar ways of relational thinking, are not simply a global geographical difference but are already present in African urban environments themselves (and indeed to some extent in Western urban environments). While African people who used to live in rural environments and move to the city or to the diaspora (US, Europe, etc.) might maintain a sense of “I am because we are”, keep a sense of belonging to their culture and community, and often stay in touch with their rural relatives or relatives in Africa, for example by using digital technologies such as WhatsApp and other social media (which mitigates the confusion and alienation Mbiti talks about), they also become part of a (more) individualistic environment. And individualism is also a culture and a form of life. In the West and in the African cities, people are not a-social or a-cultural but are *also* embedded in a form of life–albeit one that encourages them to think of themselves as individuals who are *not* related in the ways described by Ubuntu scholars. In that Wittgensteinian sense, we are always relational, whether we recognize it (for example in Ubuntu) or not. And human society is only possible if there is a network of relations (Ogude, 2018). Furthermore, urban people–in Africa and elsewhere–have their own rituals, games, and rules that help them to cope in an insecure world. In the West and in the cities, people are taught that community is not primary and that it is their own responsibility to take care of themselves. Taking care of the other is not the most important duty but needs justification. Although there is variety in modern Western ethics and political philosophy and indeed within Western culture, which is not homogenous and sometimes includes relational aspects too (consider for example the US versus European societies, or utilitarianism versus virtue ethics, which can and has been developed in communitarian ways, for example in the work of MacIntyre and Taylor) and while dichotomies such as East–West, North–South etc. are a helpful heuristic but have their limitations, the starting point in the West and in modern, westernized urban environments is usually individuals and their interests. This individualism fails to recognize the basic relational nature of persons and human societies, and promotes a view of society that threatens to render society itself impossible (in the U.S., but also elsewhere, we see clear signs of this). Ubuntu philosophy reminds everyone, also and especially in the West, about the truth and value of connectedness and interdependence.

What does this use of Wittgenstein and this discussion of Ubuntu mean for the use and development of social robotics? What social robots are and become will depend on the cultural context, on the form of life that robot is meant to function in. If the form of life is Western and individualistic, then robots are designed for individuals, for example when they are meant to function as companions for urban people who feel lonely; or as care workers for individuals in their isolated homes. When community and solidarity are lacking and without a truly relational perspective, the “social” in social robot means that it is designed to interact with individuals in order to respond to their unique, individual interests, needs, wishes and commands and to support their human dignity understood as individual autonomy. The human is the master and the robot has to focus on that individual as such. It has to serve and keep “company”. The cultural background is an atomistic conception of the person and a society and technologies that risk creating loneliness and isolation. In the West robots are embedded in such a culture and may contribute to such a society in so far as they are meant to replace human companions (Turkle, 2011). I say “in so far” since social robots are also often meant to facilitate contact or to provide care, without replacing humans. But even in those cases, the robots are designed to support the individual. Sometimes robots may be seen as a threat to the individual. But the individual is the reference point and the starting point.

If, on the other hand, we take an Ubuntu environment (or want to stimulate and create one), we have a very different starting point and hence need a different kind of “social” robot. We need a robot that is primarily focused on the interests of the family, the group, and the community. Western robots created to cater to individual needs such as companion robots and robots designed to relieve loneliness are unlikely to be accepted in a cultural environment (for example an Ubuntu one) in which there is still a sense that companionship can be found in the community. An “Ubuntu robot” would need to align with, and preferably support, this Ubuntu *common sense*: the sense that human beings are always part of a community and (other) things that are larger than ourselves. And the robot itself, to the extent that it is social at all, is not so much an “individual” but a member of the group. It needs to fit into the Ubuntu way(s). In analogy to the humans who grow up in Ubuntu communities, the robot will have to learn how to play the games of the community and fit into the form of life of that particular community. It will have to be given a particular social role within the community and receive its goals not from particular individuals (qua individuals) but–via individuals perhaps–from the group and the community. When the robot does something, it will do it not because “I want” and “I desire” it to do something for me, but because *we* think it is what has to be done. The robot will support humans, but not because they are individuals but because they are part of the group and the group needs to be supported and its future needs to be secured. If a robot is threatening at all in this case, it is so because it is a threat to the community and its way of life.

This different normative direction thus changes the meaning of the robot. Technically speaking, it may well be the same artefact. But the way it is used, is programmed, and learns will tap into different games and will be integrated in a different, less individualist form of life. In this analysis, we do not focus on the robot-object but about the meaning it gets through its use and development in a particular context. The robot becomes an “Ubuntu robot” not just and not mainly by getting different hardware or software, but by being used, behaving, and learning in a different–Ubuntu–interactive context with its different games and form of life. (In that sense, the shorthand term “Ubuntu robot” itself might be misleading, as it might be interpreted as putting the emphasis on an individuated technological object rather than (the relation between that artefact and) the culture. Instead, what one may call “Ubuntu-informed robotics” is about the robot being embedded in a particular cultural and social context, being part of particular games. In both cases, however, our language cannot very well capture the dynamic and interactive relation between technology and culture.)

At the same time, social robots may also be used in ways that change the (rules of the) game. A particular ritual may be carried out slightly differently when a robot participates in it. Consider for example a robot conducting a marriage ritual: likely, this will not be exactly the same as when a human does it. Or consider again the more mundane ritual of coffee or tea: robots will likely make small changes to the way it is done. And these changes can be and should be evaluated, for example by abstract Western moral philosophy or by a particular community. Adopting a more cultural and more relational perspective does not mean that anything goes. That would be normative, ethical relativism, according to which ethics is seen as relative to the norms of the society and culture in which it is practiced. It does mean, however, that we recognize cultural variety at the descriptive level: in different societies and cultures, there *are* different ethical practices and norms, and there are different meanings and different ways of doing things. It means that we recognize, for example, that often the robots we talk about in philosophy of robotics are Western robots and urban robots, and that this gives us a limited perspective on robotics and also limits our imagination of the robotic future. The current neglect of African philosophy in intercultural thinking about robotics creates such a limitation. More generally: it implies that we recognize that social robots and their meaning is always already unavoidably linked to particular games and a particular form of life. Rather than opening the door for ethical relativism,^3^ this insight enables us to become aware of the cultural embeddedness of robots and to take critical distance from our own (in my case and that of many readers: Western) perspective on what social robots are and might become. And ultimately, if Pannikar is right: it also means to take critical distance, and potentially re-imagine, our own cultural perspective on what we are and might become.

## Who Needs an Ubuntu Robot?

Finally, one may ask whether we really need robots at all in order to promote the relational Ubuntu perspective on the social. One may object, for example, that Africa does not need robots and other high tech but (low tech and political) solutions to more urgent problems such as lack of clean water and droughts. Culturally sustainable robotics might seem a Western hobby. And is it, in the light of climate change and environmental problems, not more important to think about *ecologically* sustainable robotics? One could also argue that robots are often used in the context of capitalist exploitation; the Ubuntu robot might be misused to continue these forms of exploitation. And one could say that instead of technology we need human beings who are educated in a more relational way and act in a more relational way. There is truth in all this. However, the emphasis in this essay is not on developing social robots for and in Africa, but on developing an Ubuntu-inspired philosophical perspective on social robotics, understanding the relation between technology and culture, and conceptualizing technology’s role in catalyzing social change. Furthermore, one could respond that one should avoid stereotypes about Africa (for example, one may remark that there is also socio-economic and not only cultural diversity in Africa, that there is also a need for high tech in Africa, and that there is also research and innovation in Africa^4^), that high tech and low tech can go hand in hand, that both ecological catastrophe and capitalist exploitation go against any truly relational Ubuntu perspective and values, and that there is no dilemma between having Ubuntu robots and Ubuntu people.

Yet the answer to the question who needs “Ubuntu robots” most (in the sense of the ideas and approach developed in the paper, not just the artefacts: the question is who needs to change accepted ways of thinking by drawing on Ubuntu), is probably not Africa but the West. It seems that the West stands most in need of the values embodied in Ubuntu cultures and traditions, and is generally open to new technologies as a medium for cultural understanding and cultural change. Ubuntu robots and other Ubuntu technologies and perspectives may infuse Ubuntu values into the Western perspective and thus contribute to harvesting the opportunities and coping with the challenges of modernity – which, after all, is still a relatively short social and political experiment viewed from the perspective of the history of humanity. The idea is not that the West should return to traditional forms of life (its own historical ones, African ones, or others) or mine and expropriate Ubuntu in a colonial and romantic way, using “exotic” Ubuntu to spice up its own culture that is understood as boring or declining, but that it can learn from other cultural perspectives in order to better understand and adapt its own form of life in the light of problems that show the limitations of its own perspective, for example the limitations of individualist thinking and individualist society in the light of ecological and climate challenges. This learning and this change is gained through the kind of intercultural dialogue mentioned in the introduction, and technology can assist with this risky but necessary project. Through the technology, that is, through the way it is used in particular contexts, Ubuntu values such as care for others and community solidarity can help people in the West to reflect on their own culture and inspire cultural change in non-Ubuntu cultures (and the same is true, of course for other values drawn from other non-Western cultures, including Eastern ones; the Ubuntu perspective offered here is not meant to be exclusive or competitive in any way). Through a particular use and in the appropriate kind of context, social robots can assist this cultural exchange and cultural learning.^5^

This role for social robots brings out again both the hermeneutic dimension of technology and the culturally creative, game changing role of technology. First, it makes clear again that technologies such as robots are not just about functioning and getting things done but also about meaning and culture. Second, robots can also contribute to new meaning-making and to changing games and ways of life. The point of culturally sustainable robotics should not mainly be to embed robots in an existing culture and context, say in Ubuntu communities in Africa, for example in order to increase their acceptability and enable their full functioning in the rich, cultural sense I have proposed in this paper. The point should also be to use technologies (next to other means) to help us reflect on, and change, that very culture–in Africa, in the West, and elsewhere.^6^ In principle this technology-enabled exercise may happen through commercial technological products, but it can also be done through educational programs and through art, including art that uses robots. For example, the concept of the Ubuntu robot could be developed into an art project that uses robots in ways that link robotics to particular games and a particular form of life: that brings out this particular form of life (puts it on display, shows it, demonstrates it), for example Ubuntu, and encourages us to critically reflect on our own (Western) form of life and, if necessary, change it. Sustainability does not mean absence of change; on the contrary, only through change and through being lived can cultures persist.

Note that social robots cannot play a role in this critical project as mere symbols or as icons, as Sparrow (2020) has argued. It is true that robots convey meanings. But the use of the term icon suggests that they do so as objects linked to representational content. If the Wittgensteinian framework outlined above gets it right, however, this function would be analogous to Wittgenstein’s word-objects and needs to be criticized: use, not representation, is central. Social robots can contribute to cultural sustainability only by being used. Only through use they can gain sufficient meaning to support sustaining and critical evaluating a culture. Like culture, technology needs to be performed. Normatively speaking, this emphasis on use also means that the users should not remain out of view: any project of cultural sustainable robotics can only work if it is supported by people: not only the developers, designers, artists, and other directly involved in the process, but also other stakeholders. The Ubuntu robot project, for example implemented as an art and science project, can work only if people are open to engaging with the robot and the questions it poses. And ideally people from Ubuntu philosophy and cultures should have a say in the interpretation of Ubuntu offered by the (makers of the) technology. For example, if generalization and simplification about Ubuntu are seen as problematic or if stereotyping is detected and found potentially insulting or oppressive, then the challenge is to constructively do something about that in a particular context together with those (potentially) affected. Participatory design and public engagement methodologies, which are already used in social robotics, may be helpful here. Participation and design starting from “we” rather than “I” is also Ubuntu. Furthermore, the picture of the Western view (in robotics but also in intercultural philosophies) may be seen as overgeneralizing and simplifying; more work may be necessary to better integrate various relational perspectives that are already part of the Western philosophical tradition, such as feminist and ethics of care approaches, deep ecology, and posthumanism. And in the end, changing Western culture and changing technologies should also be common, public projects. It should not be left to a few politicians, experts, or billionaires. It is *our* problem and *our* responsibility; it is, literally, a *communicative* matter. This encourages us to ask political questions regarding the organization and ownership of social robotics, and, more generally, the high-tech sector.

However, to make sense of this claim about technologies and communication we need a richer understanding of the concept of communication. As James Carey (1992: 14–22) has helpfully shown in his *Communication as Culture*, communication can be understood as the transmission and transportation of messages (the transmission view), but also as ritual (the ritual view): the latter does not concern exchanging information and data, but the non-computable experience of sharing and of what David Gunkel and collaborators call the coming ‘into contact with one an-other’ (Gunkel et al., 2016: 12). This has to do with commune and community (Carey, 1992: 18). Communication is then the result of, and constitutive of, community. It is about the construction, maintenance, and (I add) transformation of the cultural world. This opens up a more social perspective on culture, cultural change and communication and one that is more relational and more “Ubuntu” than that provided by Western individualism.

## Conclusion: Towards a Post-Individualist Culture?

In this paper I have offered a framework for thinking about the relation between social robots and culture and I have explored what it would mean to use and develop a non-Western, in particular Ubuntu robot. This has resulted in directions for an interesting relational view on the person that goes against Western individualism. It also has revealed the role technology can play not only to affirm and perpetuate a particular form of life but also to philosophically examine it, question it and potentially change it. For technology development and technology use in general, this work contributes to a more diverse and open perspective that recognizes (1) that there are different ways of doing things (different games and forms of life) in different cultural contexts, (2) that in so far technology is a matter of use, its meaning and functioning are intrinsically connected with these contexts and (3) that technology can and should reflect this and respond to all this diversity and semantic-pragmatic possibilities: that we can and should develop technology in different ways, ways that not only help technology to be embedded and accepted in particular contexts (e.g. particular African contexts) but also contribute to projects of cultural hermeneutics and social and cultural change.

For culturally sustainable robotics, this implies that we have to recognize that the definition and evaluation of the cultural sustainability of particular social robots will depend on how they are (or might be) used in a particular context, game and form of life and what they do or might do to that game and form of life (shape it, reveal it, change it, etc.). And in the end, cultural sustainability of robotics will also depend on our understanding and evaluation of the sustainability of that culture and form of life itself and our understanding and evaluation of how they evolve. It is questionable, for example, whether Western individualist culture can offer sufficient resources to sustain itself as a form of life. It may well be that South and East Africa are post-Ubuntu now, to the extent that their cultures are already Westernized. While, as I indicated, elements of traditional Ubuntu remain, even in urban environments in Africa, like all cultures and forms of life, Ubuntu and African cultures are dynamic rather than fixed and should not be romanticized. But should the West also change and move to post-individualism? Is it already doing so or not really? What, precisely, is the nature and structure of the current Western forms of life, and how sustainable are they? Do Western people need different rituals, games, and performances? Which new ones are already emerging? What can the West learn from African forms of life that are situated on a continuum or spectrum between tradition (for example Ubuntu) and Western individualism? And what role can technologies such as robotics play in this? How can technologies help us (Westerners but also others) to deeply interrogate our own culture? How can they help to communicate, that is, how can they help to make community? How can they help us to gain more insight into the relational nature of reality, and what does this relationality imply for our lives and for the way we should organize our societies? These are important questions for the West, but also for the Westernized cultures and modern urban centers of the African continent. Our futures and indeed our common future depends on it.

## Footnotes

1 Keeping in mind recent developments in information ethics, note that this relationality is not the end point (hen) of Ess’s ethical theory-driven pros hen pluralism or the alternative end point (empirical social reality) Hongladarom’s (2021) proposes in response to Ess (2020), but rather a kind of starting point or ground, a condition of possibility for dialogue, which is not reducible to conceptual connections or social agreement. For recent research and thinking on how intercultural dialogue is possible and on the problem of translation, see for example Seibt and Garsdal (2015).

2 Compare also with social construction of technology (SCOT) approaches (for example in the work of Bijker and Pinch)– however, I will not do this here.

3 My rejection of both ethical absolutism and ethical relativism is in line with the pluralist and ‘relationist’ positions defended respectively by Charles Ess (2006; 2020) and Luciano Floridi (2013: 32–33), and also with Deweyan pragmatism and perhaps Pannikar’s hermeneutics.

4 Such claims would of course need further empirical support.

5 In addition, and as is recognized by many scholars in philosophy and theology of social robotics (see for example Foerst interviewed in Dreifus 2000 or Coeckelbergh 2022), they also can help us to reflect on what it means to be human – which again is an exercise that is done in a particular cultural context, preferably in dialogue with a variety of traditions within and outside that context.

6 This also includes interrogating our basic assumptions about what it means to be human and how we do and should relate to human and non-human others, starting from within a specific cultural context.
